# Effect of medically lowering intraocular pressure in glaucoma suspects with high myopia (GSHM study): study protocol for a randomized controlled trial

**DOI:** 10.1186/s13063-020-04748-7

**Published:** 2020-09-29

**Authors:** Feng Bin Lin, Shi Da Chen, Yun He Song, Wei Wang, Ling Jin, Bing Qian Liu, Yu Hong Liu, Mei Ling Chen, Kai Gao, David S. Friedman, Jost B. Jonas, Tin Aung, Lin Lv, Yi Zhi Liu, Xiu Lan Zhang, on behalf of the GSHM study group, Xiu Lan Zhang, Yi Zhi Liu, Lin Lv, David S. Friedman, Jost B. Jonas, Tin Aung, Shi Da Chen, Wei Wang, Feng Bin Lin, Yun He Song, Fei Li, Kai Gao, Bing Qian Liu, Yu Hong Liu, Mei Ling Chen, Neil M. Bressler, Ki Ho Park, Ming Guang He, Ching-yu Cheng, Paul Healey, Xiang Chen, Guang Xian Tang, Ling Jin

**Affiliations:** 1 State Key Laboratory of Ophthalmology, Zhongshan Ophthalmic Center, Sun Yat-sen University, No.7, Jinsui Road, Guangzhou, 510060 China; 2 Massachusetts Eye and Ear, Harvard University, Boston, USA; 3 Department of Ophthalmology, Medical Faculty Mannheim, Heidelberg University, Heidelberg, Germany; 4 Singapore National Eye Center, Singapore, Singapore

**Keywords:** Glaucoma suspect, High myopia, Intraocular pressure, Randomized controlled trial

## Abstract

**Background:**

Currently, whether and when intraocular pressure (IOP)-lowering medication should be used in glaucoma suspects with high myopia (GSHM) remains unknown. Glaucoma suspects are visual field (VF) defects that cannot be explained by myopic macular changes or other retinal and neurologic conditions. Glaucoma progression is defined by VF deterioration. Here we describe the rationale, design, and methodology of a randomized controlled trial (RCT) designed to evaluate the effects of medically lowering IOP in GSHM (GSHM study).

**Methods:**

The GSHM study is an open-label, single-center, RCT for GSHM. Overall, 264 newly diagnosed participants, aged 35 to 65 years, will be recruited at the Zhongshan Ophthalmic Center, Sun Yat-sen University, between 2020 and 2021. Participants will be randomly divided into two arms at a 1:1 ratio. Participants in the intervention arm will receive IOP-lowering medication, while participants in the control arm will be followed up without treatment for 36 months or until they reach the end point. Only one eye per participant will be eligible for the study. If both eyes are eligible, the eye with the worse VF will be recruited. The primary outcome is the incidence of glaucoma suspect progression by VF testing over 36 months. The secondary outcomes include the incidence of changes in the optic nerve head morphology including the retinal nerve fiber layer, and retinal ganglion cell-inner plexiform layer loss, progression of myopic maculopathy, visual function loss, and change in the quality of life. Statistical analyses will include baseline characteristics comparison between the intervention and control groups using a two-sample *t*-test and Wilcoxon rank sum test; generalized linear models with Poisson regression for the primary outcome; Kaplan-Meier curve and log-rank test for the incidence of the secondary outcome; and longitudinal analyses to assess trends in outcomes across time.

**Discussion:**

To the best of our knowledge, the GSHM study is the first RCT to investigate the impact of medically lowering IOP in GSHM. The results will have implications for the clinical management of GSHM.

**Trial registration:**

ClinicalTrials.gov NCT04296916. Registered on 4 March 2020

## Administrative information

Note: the numbers in curly brackets in this protocol refer to SPIRIT checklist item numbers. The order of the items has been modified to group similar items (see http://www.equator-network.org/reporting-guidelines/spirit-2013-statement-defining-standard-protocol-items-for-clinical-trials/).

**Table:** 

Title {1}	Effect of medically lowering intraocular pressure in glaucoma suspects with high myopia (GSHM study): study protocol for a randomized controlled trial
Trial registration {2a and 2b}.	ClinicalTrials.gov, NCT04296916. Registered on 4 March 2020, https://register.clinicaltrials.gov
Protocol version {3}	22/8/2020, version 2.0
Funding {4}	High-level Hospital Construction Project, Zhongshan Ophthalmic Center, Sun Yat-sen University (Funding number: 303020104)
Author details {5a}	^1^ State Key Laboratory of Ophthalmology, Zhongshan Ophthalmic Center, Sun Yat-sen University, Guangzhou, China ^2^ Massachusetts Eye and Ear, Harvard University, Boston, USA ^3^ Department of Ophthalmology, Medical Faculty Mannheim, Heidelberg University, Heidelberg, Germany ^4^ Singapore National Eye Center, Singapore, Singapore
Name and contact information for the trial sponsor {5b}	No sponsor
Role of sponsor {5c}	Not applicable

## Introduction

### Background and rationale {6a}

Myopia has emerged as a major health issue in East and Southeast Asia, especially the sight-threatening complications associated with high myopia (HM). In 2000, it was estimated that there were 163 million people with HM with a projected increase to almost one billion by 2050, corresponding to 2.7% and 9.8% of the world population, respectively [1]. The prevalence is even higher in Asian countries. In China, the proportion of highly myopic teenagers is between 11.1 and 19.5% [2–4]. Complications of HM can be associated with significant ocular morbidities, including maculopathy, retinal detachment, and glaucoma.

HM is associated with an increased prevalence of glaucoma. Population-based studies indicated that individuals with myopia have an approximately doubled risk of developing open-angle glaucoma in comparison with those without myopia and that the odds ratio was 5.90 in eyes with HM [5, 6]. The risk for glaucomatous optic neuropathy (GON) increases with longer axial length in a non-linear manner. A hospital-based study in Tokyo reported that 27.3~28.5% of HM patients had GON [7, 8]. However, accurate diagnosis of glaucoma in HM is a challenge, since classic glaucomatous changes in an HM eye are often difficult to detect. First, HM can cause tilted optic discs and large peripapillary atrophy obscuring the disc edge and shallow cupping, making the detection of glaucomatous optic disc damage difficult. Second, myopic degeneration of the macula can also mimic glaucoma visual field (VF) defects [9]. Moreover, intraocular pressure (IOP) in patients with HM and glaucoma is generally within the normal range [7]. Therefore, due to the lack of a uniform diagnostic standard, more and more viewpoints have been raised that HM eyes with optic disc head damage and/or VF defects should be classified as glaucoma suspects.

IOP is the only modifiable parameter in glaucoma and glaucoma suspect patients [10]. However, the decision to begin treatment to lower the IOP in the glaucoma suspects is complex, especially for glaucoma suspects with HM (GSHM). The effect of IOP lowering on GSHM is controversial [11–13], although animal studies demonstrated that topically applied latanoprost is associated with the reduction in the progression of myopia [14]. Our observational studies also found that IOP lowering could slow the progression of GSHM (unpublished data), although the sample sizes were small and of relatively short follow-up periods. Therefore, randomized trials are required to evaluate whether IOP lowering influences the incidence of glaucoma suspect progression in HM eyes. In this study, we report the design and methodology of a randomized controlled trial (RCT) designed to test this hypothesis.

### Objectives {7}

The main aim of the GSHM RCT is to test the hypothesis that medically lowering the IOP by 20% from the baseline would reduce the incidence of glaucoma suspect progression in HM eyes by VF testing compared with simple follow-up over a 36-month observation period. We will also evaluate the association of medically lowering IOP with the incidence of changes in the optic nerve head morphology, including the retinal nerve fiber layer (RNFL), retinal ganglion cell-inner plexiform layer (GCIPL) loss, progression of myopic maculopathy, loss of visual function, and change in the quality of life.

### Trial design {8}

**Fig. 1:**
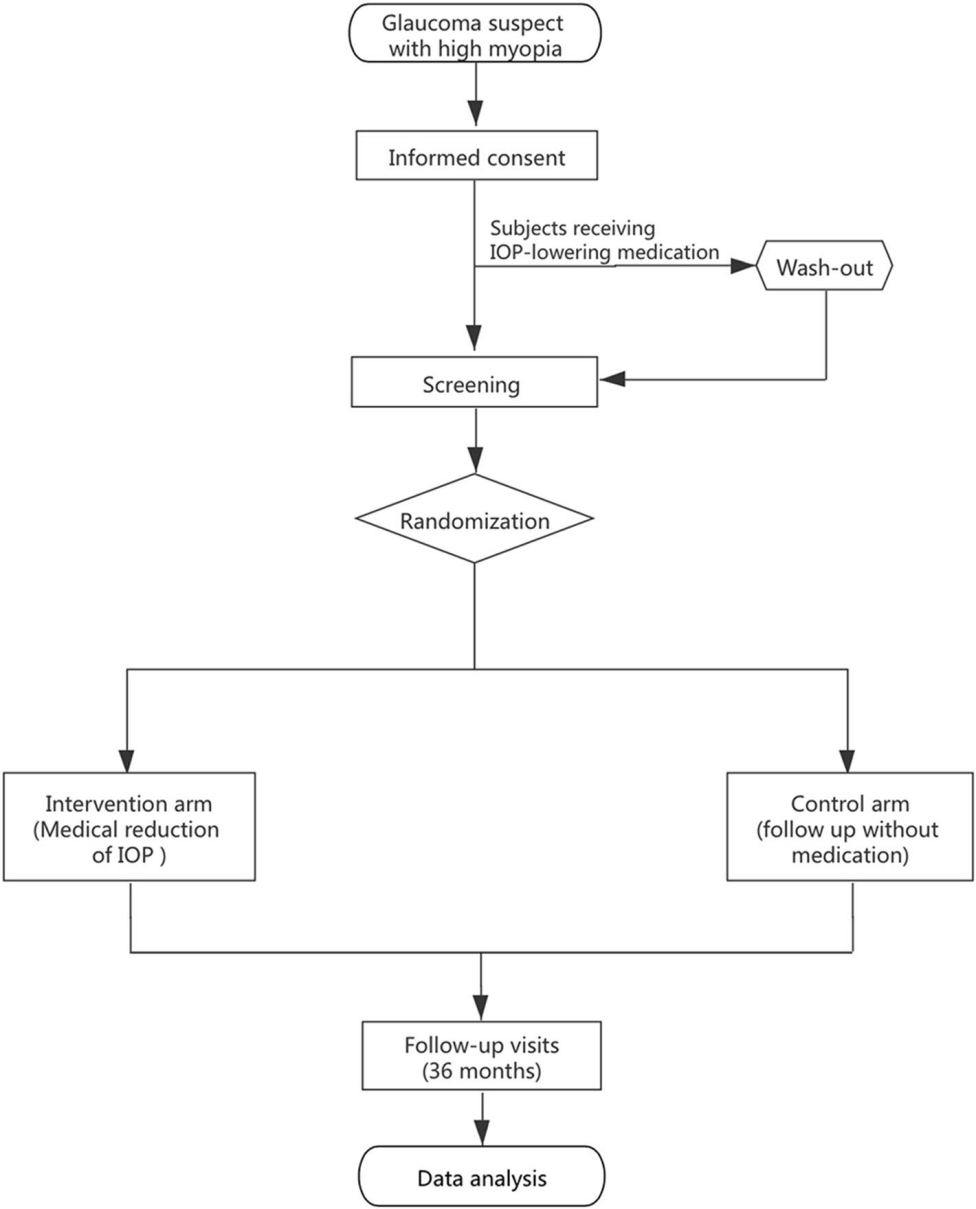
Schematic of the GSHM study design

The GSHM study is an open-label, single-center, RCT for GSHM. Eligible participants will be randomized to either receive topical IOP-lowering medication to achieve IOP reduction of 20% as compared to the baseline or simple follow-up without treatment, as a control in a 1:1 ratio, for a study period of 36 months or until the end point is reached. Figure 1 summarizes the trial design with the details of the GSHM study.

## Methods: participants, interventions, and outcomes

### Study setting {9}

The study will be conducted at the Zhongshan Ophthalmic Center (ZOC), Sun Yat-sen University, a tertiary specialized hospital in Guangzhou, China. All examinations and interventions will be carried out at the Clinical Research Center at ZOC.

### Eligibility criteria {10}

Glaucoma suspects are defined by VF defects (see details in the section on “Plans for assessment and collection of outcomes {18a}”), which cannot be explained by myopic macular changes, or other retinal and neurologic conditions. Only one eye per participant will be eligible for the study. If both eyes are eligible for the study, the eye with the worse VF (worse mean deviation [MD]) will be recruited. If the MD is the same in both eyes, then the eye with the better best corrected visual acuity (BCVA) letter score will be included.

#### Inclusion criteria

- Age between 35 and 65 years.
- Diagnosed with HM (spherical equivalent ≤ − 8.00 diopters or axial length ≥ 26.5 mm) [15, 16].
- Diagnosed with glaucoma suspects [10], which cannot be explained by myopic macular changes, or other retinal and neurologic conditions.
- IOP ≥ 12 mmHg and ≤ 24 mmHg on at least two visits, as measured by the Goldmann applanation tonometry [13].
- An open anterior chamber angle based on gonioscopy.
- BCVA ≥ 6/12.

#### Exclusion criteria

- Allergy to prostaglandins.
- Advanced VF loss (MD worse than 16 dB) or a threat to fixation (sensitivity 10 dB or worse affecting either or both test points closest to the point of fixation in the upper hemifield and at either or both of the corresponding test points in the lower hemifield) in either eye [17].
- Previous IOP-lowering surgery in the study eye (i.e., trabeculectomy, Ahmed glaucoma valve implantation, any laser trabeculoplasty).
- Previous cataract surgery in the study eye.
- Previous corneal refractive surgery in the study eye.
- Clinically significant or progressive retinal disease such as proliferative diabetic retinopathy, retinal detachment, central retinal vein occlusion, or retinitis pigmentosa in the study eye.
- Chronic, recurrent, or severe inflammatory eye disease in the study eye (from screening), such as chronic or recurrent uveitis.
- Obvious corneal and iris lesions, severe cataracts interfering with fundus examinations, or monophtalmia.
- Need for ocular surgery/laser or the anticipated need for cataract surgery that would influence the ophthalmological parameters measured in this study during the study period.
- Other serious systemic diseases (i.e., hypertension, heart disease, diabetes, or rheumatic immune system diseases).
- Pregnant or nursing women.

### Who will take informed consent? {26a}

Participants meeting the eligibility criteria will be invited to participate in the study. The investigator will explain the nature and purpose of the study, and inform the participant of the medication and examinations to be performed. The risks of participation and visit schedules will also be explained. Opportunities will be given to the participants to ask questions about the study. Written consent will be obtained from participants who agree to be enrolled into the study in the presence of a witness. The consent will be obtained in the privacy of the consultation room, and participants will not be restricted to a time limit within which to decide whether they would like to participate or not.

### Additional consent provisions for collection and use of participant data and biological specimens {26b}

On the consent form, participants will be asked if they agree to the use of their data should they choose to withdraw from the trial. Participants will also be asked for permission for the research team to share relevant data with people from the universities taking part in the research or from regulatory authorities, where relevant. This trial does not involve biological specimen collection for storage.

## Interventions

### Explanation for the choice of comparators {6b}

The choice of the comparator is based on the current GSHM treatment in clinical.

### Intervention description {11a}

Participants will be randomly divided into two arms in a 1:1 ratio.

**Fig. 2:**
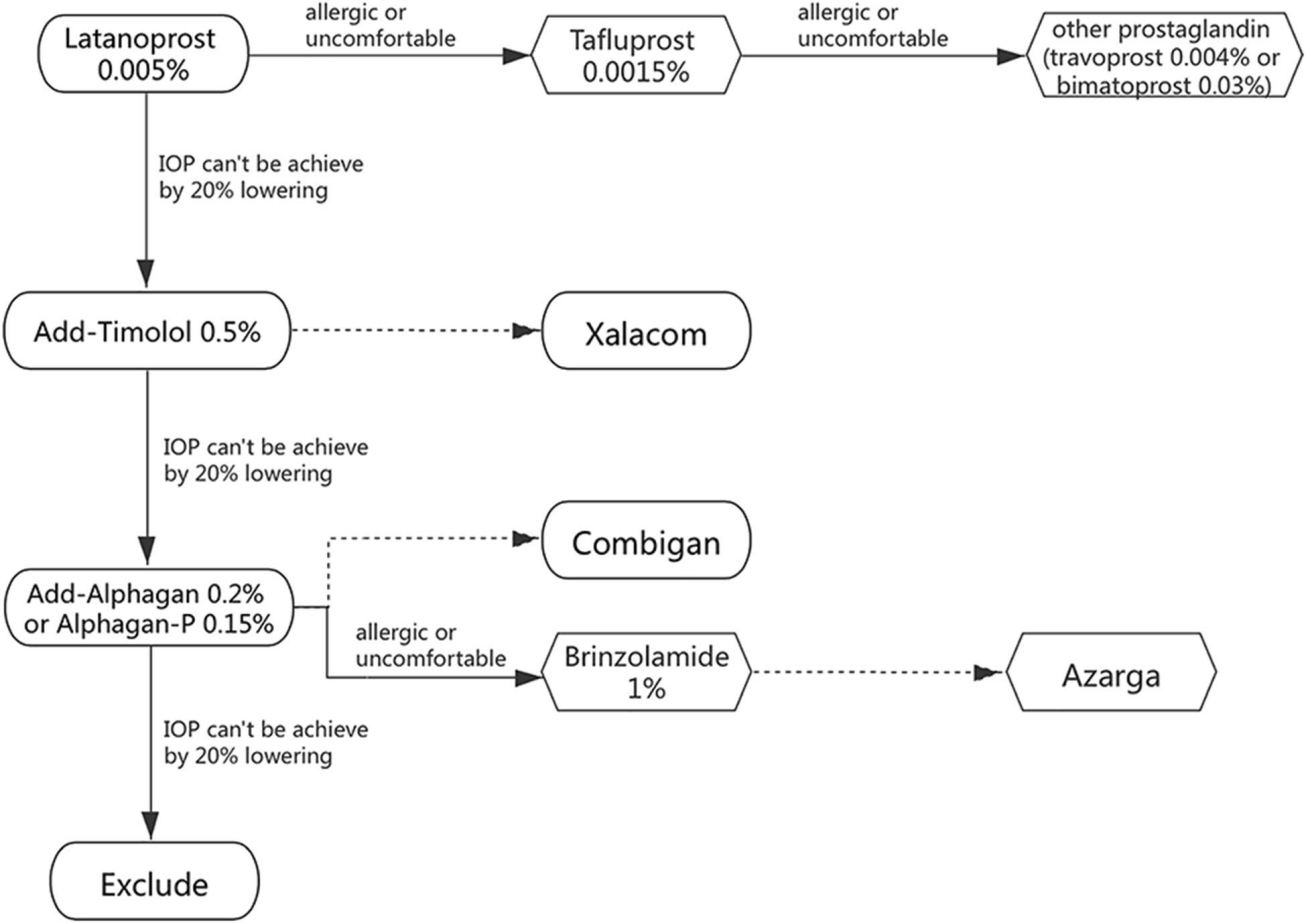
The schematic of the intervention design

Participants in the intervention arm will receive medical treatment to reduce of the IOP for 36 months or until they reach the end point (Fig. 2). Latanoprost 0.005% eye drops will be the first choice for treatment. If an individual is allergic to latanoprost or feels uncomfortable with it, the eye drops will be switched to tafluprost 0.0015% eye drops, or other prostaglandin eye drops (travoprost 0.004% or bimatoprost 0.03%). If an IOP reduction of 20% is not achieved within 3 months, timolol 0.5% will be added as a second medication. If necessary, latanoprost 0.005% and timolol 0.5% will be switched to Xalacom eye drops (a fixed latanoprost and timolol combination). If an IOP reduction of 20% is still not achieved, Alphagan 0.2% or Alphagan-P 0.15% will be added. If necessary, timolol 0.5% and Alphagan 0.2% eye drops (or Alphagan-P 0.15%) will be switched to Combigan eye drops (a fixed Alphagan and timolol combination). If an individual is allergic to Alphagan (or Alphagan-P) or feels uncomfortable with it, the eye drops will be switched to brinzolamide 1% eye drops. If necessary, timolol 0.5% and brinzolamide 1% eye drops will be switched to Azarga eye drops (a fixed brinzolamide with timolol combination). The effect of IOP lowering will be assessed 1 week after applying each medication until the target IOP was achieved, and follow-up will be conducted at the scheduled time. If an IOP reduction of 20% is not achieved, the individual will be excluded from the study.

The treatment will comprise one drop of prostaglandin ophthalmic solution in the study eye once daily in the evening if using latanoprost, tafluprost, travoprost, bimatoprost, or Xalacom, and one drop in the study eye twice daily if using timolol, Alphagan (or Alphagan-P), Combigan, brinzolamide, or Azarga.

Participants in the control arm will be simply followed up without treatment for 36 months or until they reach the end point.

### Criteria for discontinuing or modifying allocated interventions {11b}

The end points of the study will be the characterized VF progression (see details in the section on “Plans for assessment and collection of outcomes {18a}”). When an end point is reached, all therapeutic constraints are also lifted, and the participants receive treatment according to the individual clinician’s judgment.

### Strategies to improve adherence to interventions {11c}

Every month, we will send a text message or make a phone call to remind participants to regularly take the drops regularly. Furthermore, compliance with the treatment among participants in the intervention arm will be encouraged at every follow-up visit. A medication inventory and participant diary will be used to record the adherence during the trial period.

### Relevant concomitant care permitted or prohibited during the trial {11d}

Participants who were already receiving IOP-lowering medication will need to be eluted for different periods, as follows: 4 weeks for prostaglandin analogs, 3 weeks for beta-blockers, 2 weeks for adrenalin agonists, and 5 days for cholinergic agonists and carbonic anhydrase inhibitors.

### Provisions for post-trial care {30}

The investigator provides insurance services for the participants in the clinical study in accordance with the necessary procedures described in the Good Clinical Practices (GCP). Injuries and compensations related to the clinical research will be judged by the data monitoring committee (DMC) and conducted in accordance with the applicable laws and regulations in China.

### Outcomes {12}

The primary outcome is the incidence of glaucoma suspect progression within 36 months. The progression is defined by the VF progression (see details in the section on “Plans for assessment and collection of outcomes {18a}”).

The secondary outcomes include the incidence of changes in the optic nerve head morphology including the RNFL and GCIPL loss, progression of myopic maculopathy [18, 19], loss of visual function (NEI-VFQ-25), and change in the quality of life (EQ-5D-5L).

### Participant timeline {13}

**Fig. 3:**
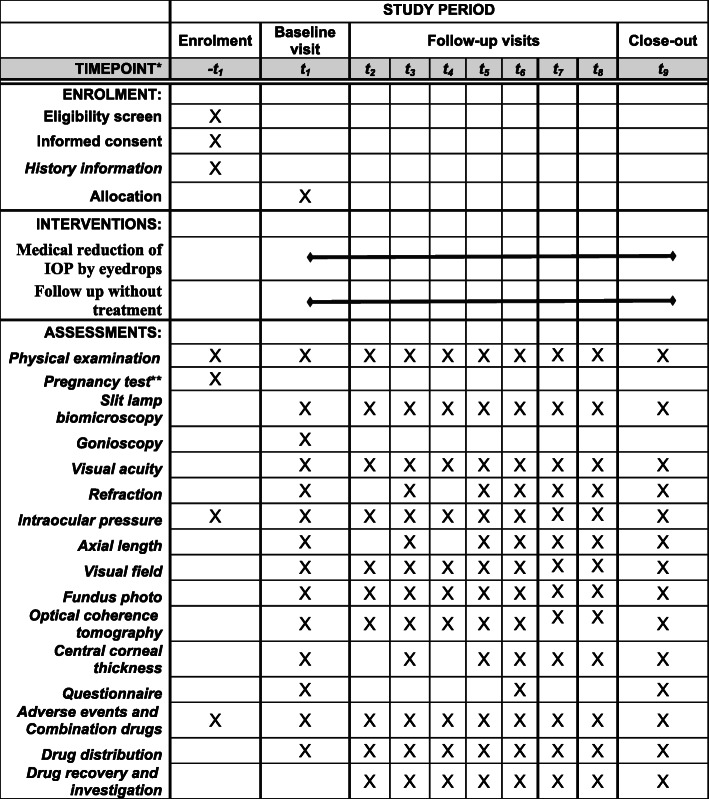
The schedule of enrollment, interventions, and assessments

The time schedule for enrollment, interventions, assessments, and visits for the participants is summarized in Fig. 3.

### Sample size {14}

The sample size calculation was based on the primary outcome, the incidence of glaucoma suspect progression over 36 months. The hypothesis was formed according to the findings of the relevant publications. The Early Manifest Glaucoma Trial (EMGT) [17, 20] reported that the 2-year progression rate among patients with early primary open-angle glaucoma was 24% in the non-medication group and 11% in the medication group. The United Kingdom Glaucoma Treatment Study (UKGTS) [21, 22] reported that the 2-year progression rate of patients with early and middle primary open-angle glaucoma was 25.6% in the placebo group and 15.2% in the IOP-lowering medication group. The risk of progressing to open-angle glaucoma among patients with HM was nearly five times that of the non-myopia individuals [5, 6]. In this study, it is expected that the IOP-lowering medication can reduce the 36-month progression rate of GSHM by 18%, assuming an incidence of 30% in the control group. At a two-sided significance level of 0.05, in order to achieve 90% power, 106 participants will be required in each study group. Considering a 20% of attrition rate over 3 years, the final sample size will be 132 participants per group and a total 264 for the two groups [23–27]. The sample size was calculated using PASS 11.0 software (NCSs, LLC, Kaysville, UT, USA).

### Recruitment {15}

Consecutive potentially eligible participants will be identified at the ZOC. Potentially eligible participants will attend a training visit where the VF test will be performed. Participants meeting the diagnostic criteria of GSHM criteria will be enrolled in this randomized trial.

## Assignment of interventions: allocation

### Sequence generation {16a}

Block randomization will be used to avoid distribution bias. Qualified participants in each block with a size of six will be evenly (1:1) assigned to the intervention group and the control group. After all inclusion and exclusion criteria are confirmed, a written informed consent form will be signed. The random sequence will be generated by an electronic data collection (EDC) system.

### Concealment mechanism {16b}

The allocation list will be imputed and stored in the EDC system. The sequence list will not be accessible to the investigators. Upon randomization, an A or B representing the treatment and control groups will be assigned and identified by EDC system. The groups represented by A and B will be recorded and saved in a letter.

### Implementation {16c}

Participants will be consecutively enrolled by the investigators. The participants will be allocated randomly to the intervention or control group by the EDC system.

## Assignment of interventions: blinding

### Who will be blinded {17a}

Participants and physicians will not be blinded to the treatment assignment. The primary study outcome is based on computerized VF criteria, which include fundus photographs to be read by masked graders at the end point adjudication committee (these are three independent glaucoma fellowship-trained ophthalmologists. If two ophthalmologists disagree on the diagnosis and/or the progression of the disease, the reports will be submitted to the third ophthalmologist for further judgment). Other important variables, such as optical coherence tomography (OCT), visual acuity, and IOP, will also be obtained by masked ophthalmic technicians according to standard protocols. The masking status of the technicians collecting the data will be recorded at each study visit. The study data will be analyzed by masked researchers.

### Procedure for unblinding if needed {17b}

The design is open label, with outcome assessors and data analysts being blinded, so unblinding will not occur.

## Data collection and management

### Plans for assessment and collection of outcomes {18a}

Nine visits over 36 months are planned, with follow-up examinations scheduled at a minimal frequency of every 3 months for the first year and every 6 months thereafter.

#### Visual field testing

All tests will be performed with the Humphrey Field Analyzer Mark 3 (Carl Zeiss Meditec, Dublin, CA, USA) and the Swedish interactive threshold algorithm (SITA) standard 24-2 program.

A VF defect [21, 22], for trial inclusion, is defined as a reproducible (in at least two consecutive reliable tests) reduction in sensitivity at two or more contiguous points with a probability indicated by a *p* value of < 0.01 or more, or three or more contiguous points with *p* < 0.05 or more in the pattern deviation plot, in the superior or inferior arcuate areas; or a 10-dB difference across the nasal horizontal midline at two or more adjacent points in the total deviation plot. A reliable VF will be one with false-positive and false-negative errors of less than 15% and fixation losses of less than 20%. Reliability is based on subjective judgment, including assessment of the eye tracker trace. Unreliable tests will be repeated, either on the same day (after a break of at least 30 min) or another visit within 1 month. No more than five attempts to achieve at least two reliable VF tests will be allowed [13].

Progression analysis will be performed using the Humphrey Field Analyzer Mark 3 Guided Progression Analysis (GPA) software and defined by at least three test points showing a significant negative change (*p* < 0.05) at the same locations compared with the baseline examination in two consecutive tests by the 24-2 VF program (tentative deterioration); and two confirmation tests will be needed to satisfy the criteria [22]. The confirmation of the VFs will be performed within 1 month. The first of the two VF confirmations is termed the “progress point.”

#### Fundus photography

Two images centered on the disc under standardized and non-standardized conditions and a single central image will be taken for each eye, after dilation, using fundus cameras (KOWA, Nonmyd, WX3D, Japan; TRC-NW400, TOPCON, Japan).

Optic disc progression will be assessed by comparing of the stereo photographs taken at baseline and the stereo photographs obtained at the follow-up examination and is defined by any of the following [17, 28, 29]: (1) enlargement of vertical cup-to-disc ratio (VCDR), (2) neuroretinal rim notching (incidence or enlargement), (3) wedge-shaped RNFL defects (incidence or enlargement), or (4) disc hemorrhage, if not related to myopic changes.

#### Optical coherence tomography (OCT)

Both a swept-source OCT (DRI-OCT Triton, TOPCON, Japan) and a spectral domain OCT (Cirrus 5000 HD-OCT, Carl Zeiss Meditec, USA) devices will be used, with images acquired through dilated pupils. To ensure the quality, image quality should be higher than 60 and 5 in DRI-OCT triton and Cirrus 5000 HD-OCT, respectively.

#### Visual acuity

Visual acuity will be measured before pupil dilation, tonometry, gonioscopy, or any other technique that could affect vision. Visual acuity will be performed using an Early Treatment Diabetic Retinopathy Study (ETDRS) Logarithm of the Minimum Angle of Resolution (LogMAR) chart (Precision Vision, Villa Park, IL, USA) with standard illumination at a distance of 4 m, following the protocol used in the Refractive Error in School Children (RESC) study [30]. The BCVA will be performed using a trial frame placed and adjusted on the participant’s face based on autorefraction readings and refinement. The total score will be recorded on a data form.

#### Refraction

After pupil dilatation (with 0.25% tropicamide administered at the zeroth, fifth, and 20th min), three measurements will be obtained for each eye using an auto refractometer (KR800, TOPCON, Japan), and the average sphere, cylinder, and axis will be recorded.

#### Tonometry

Goldmann applanation tonometry (AT900, Haag Streit, Koeniz, Switzerland) will be used to measure the IOP. All participants will have three baseline IOP readings taken. These will be done between 9 and 10 am, 1 and 2 pm, and 4 and 5 pm. The mean of the three readings is respectively required to be between 12 and 24 mmHg for eligibility subjects. IOP at every follow-up visits will be measured between 9 and 11 am, or between 2 and 4 pm. The IOP would need to be checked at the same time of the day to minimize the effect of the diurnal fluctuation of IOP. The results of three consecutive measurements will be recorded at every visit. The mean of the three measurements will be used for assessment.

#### Axial length and central corneal thickness (CCT)

Axial length and CCT will be measured by IOLMaster (IOLmaster 700, Carl Zeiss Meditec).

#### Slit lamp biomicroscopy

An undilated examination of the anterior segment and gonioscopy will be evaluated using a slit lamp (BQ-900, Haag Streit, Switzerland). A dilated examination of the optic disc, macula, and peripheral retina will be carried out using a 90D indirect ophthalmoscopy lens (Ocular 90D Slit Lamp Lenses, Ocular, Washington, USA) to confirm GON and exclude any retinal pathology that is potentially sight-threatening or might require surgical intervention.

#### Questionnaires

The following questionnaires will be administered at visits #1, 7, and 13: (1) the National Eye Institute Visual Function Questionnaire-25 (NEI-VFQ-25) and (2) the registered EuroQol-5 Dimensions five-level (EQ-5D-5L).

#### Pregnancy test

Women of childbearing age would need to undergo a urine pregnancy test at the first visit to confirm that they are not pregnant.

#### Anthropometry and blood pressure

The height and weight of the participants will be obtained with shoes off using a free-standing height rod and a calibrated scale (RGZ120, Jiangsu Wujin Weighing Apparatus Factory, Jiangsu, China). At the baseline visit, blood pressure (BP) will be recorded on the left arm with the participant seated and rested for at least 5 min, using the Omron M7 Blood Pressure Monitor (Matsusaka, Mie, Japan).

### Plans to promote participant retention and complete follow-up {18b}

The goal of the study is to have as few losses to follow-up as possible. The investigator will regularly contact the participants through various methods (e.g., telephone, SMS, WeChat) during the daytime to promote participant retention and completion of follow-up.

Study participants wishing to withdraw will be asked to make a final closeout visit at which the testing described for the protocol visits will be performed. Study participants having an adverse effect attributable to a study treatment or procedure will be asked to continue follow-up until the adverse event has been resolved or stabilized.

### Data management {19}

Data collected will be recorded and keyed into the EDC system. The EDC system is secured digitally on a password-protected net server. Only the principal investigators and the study team will have access to the research data. All source documents will be stored in locked file cabinets with secure and limited access.

All researchers will be trained before data collection. Raw data will be tracked by an independent data and safety monitoring committee. For questions in the case report form, the data administrator will write a data queue request (DRQ) and send a query to the researcher through the clinical monitoring system. The researcher is to provide an answer to the data administrator as soon as possible. Data modification, confirmation, and entry will be performed, and a DRQ will be issued again if necessary.

### Confidentiality {27}

Personal information and data collected from each participant will be stored in the EDC system. Only delegated personnel in the study team will be given access to these data. In addition, the investigator(s) will permit study-related monitoring, audits and/or institutional review board (IRB) review, and regulatory inspection(s), providing direct access to source data/document. The data management team will be collecting the de-identified data for statistical analysis.

### Plans for collection, laboratory evaluation, and storage of biological specimens for genetic or molecular analysis in this trial/future use {33}

No biological specimens will be collected as part of this trial.

## Statistical methods

### Statistical methods for primary and secondary outcomes {20a}

Data will be described as mean (standardized deviation, SD) for normally distributed continuous variables, median (interquartile range, IQR) for continuous variables without normal distribution, and frequency (percentage) for categorical variables. Baseline characteristics between the intervention and control groups will be compared by performing a two-sample *t*-test and Wilcoxon rank sum test for continuous variables with and without normal distribution, respectively, and chi-square test or Fisher’s exact test for binary or multinomial categorical variables.

Generalized linear models with Poisson regression will be applied to estimate the relative risk (RR) and 95% confidence interval (CI) between groups in primary outcome, incidence of glaucoma suspect progression with HM. Secondary outcome analyses will include as follows: Kaplan-Meier curve and log-rank test to be performed for the incidence of optic disc progression, rate of RNFL and GCIPL loss, visual function (NEI-VFQ-25) score, and quality of life (EQ-5D-5L) score which will be modeled by linear regression. The progression of the myopic maculopathy grading of the fundus photographs will be analyzed using the same method as that for the primary outcome. Longitudinal analyses will also be conducted to assess trends in outcomes across time. All variables considered significant at the *p* < 0.20 by simple regression models will be included in the multiple regression model.

All statistical analyses will be performed using a commercially available software package (Stata 15, StataCorp, College Station TX, USA).

### Interim analyses {21b}

Interim analysis will not take place during this study.

### Methods for additional analyses (e.g., subgroup analyses) {20b}

Adverse events in the two study groups will be compared using the chi-square test or Fisher’s exact test. All adverse events related to the study, as well as the laboratory and clinical examinations reporting abnormalities, will be described in a table.

### Methods in analysis to handle protocol non-adherence and any statistical methods to handle missing data {20c}

The primary analysis will be performed according to the intention to treat criteria, and all missing data will be imputed. Last observation carried forward (LOCF) or multiple imputations will be used to impute missing data [31]. The multiple imputation approach creates 20 copies of the data, in which missing values are imputed by chained equations. The final results will be obtained by averaging these 20 datasets using Rubin’s rules, which ensures that the standard errors for all regression coefficients reflect the uncertainty in the imputations as well as the uncertainty in the estimation.

### Plans to give access to the full protocol, participant level-data, and statistical code {31c}

The datasets analyzed during the current study will be available from the corresponding author upon reasonable request.

## Oversight and monitoring

### Composition of the coordinating center and trial steering committee {5d}

The ZOC will be responsible for the management of the trial. A trial steering committee (SC) will be established to guarantee the quality of the study. The committee has overall responsibility and authority for directing activities, formulating policies for the study, and changing of the protocol.

### Composition of the data monitoring committee, its role and reporting structure {21a}

The DMC members include Prof. Ching-yu Cheng and Prof. Paul Healey, who have no competing interests. The responsibility of the DMC is to review the study design and study documents before the study starts of the study to identify any problems that might affect future data analysis or patient safety. They are also responsible for reviewing treatment reports prepared by the SC for evidence of adverse and beneficial treatment effects, terminating the study if treatment benefits or treatment risks are so high for one treatment group that continuation of the trial would be deemed unethical, advising the SC on interpretation of study data interpretation, and recommending to the SC changes in the study protocol based on periodic data analysis.

### Adverse event reporting and harms {22}

An adverse event is any untoward medical occurrence in a study participant, irrespective of whether the event is considered treatment related. Throughout the course of the study, all efforts will be made to remain alert to possible adverse events or untoward findings. The first concern will be the safety of the study participant, and appropriate medical intervention will be made when an adverse event occurs. All adverse events, whether voluntarily reported by the participant, or discovered by the study personnel during questioning, physical examination, or by other means, will be reported online on an adverse event form. Each adverse event form will be reviewed by the safety supervision committee to identify the required coding and the reporting actions.

Serious adverse events must be reported to the IRB, the DMC, and the Clinical Research Center and faxed to the drug registration office of the Drug Administration within 24 h, even if the adverse event is not related to the study drug. The original and fax confirmation form of the serious adverse event must be kept in the research center along with the case report form.

### Frequency and plans for auditing trial conduct {23}

The SC and the independent DMC and ethics committee will meet once a year through the trial period to review study conduct and compliance with the protocol, standard operation procedure, GCP, and the applicable regulatory requirements. The trial audit may be performed on a separate form.

### Plans for communicating important protocol amendments to relevant parties (e.g., trial participants, ethical committees) {25}

Protocol amendments will be discussed and decided by the principal investigators, SC, and DMC. The ethical committee will be notified and its approval will be sought. Deviations from the protocol will be fully documented, using a report form.

## Dissemination plans {31a}

A GSHM study publication is one that contains details of the design, methods, or results of the GSHM study and is written by the investigators. Any paper classified as a GSHM study publication must be approved by the investigators prior to submission for publication. Similarly, any presentation made on behalf of the GSHM study must be approved by the investigators. All papers of the GSHM study will be published under the conventional author format. None of the presented or published data will contain any information that will reveal the identity of the participants. Each study participant will be given a study number so that the data can be pseudo-anonymized.

As the High-level Hospital Construction Project, Zhongshan Ophthalmic Center, Sun Yat-sen University funds this study, any research results (i.e., papers, monographs, patents, appraisals, and result reports) must indicate the name of the funding body and its funding number.

## Discussion

The trial is designed to evaluate the effect of medically lowering IOP in GSHM. There are many drugs available for initial choice, including prostaglandin analogs, beta-blockers, adrenalin agonists, cholinergic agonists, and carbonic anhydrase inhibitors. Prostaglandin analogs are the most frequently prescribed initial eye drops for lowering IOP in patients with glaucoma because they are the most efficacious, well-tolerated, and instilled only once daily [22]. Moreover, it was reported that topical latanoprost could also reduce the development of myopia in guinea pigs [14]. Hence, in the GSHM study, prostaglandin analogs (latanoprost 0.005% eye drops) will be the first choice.

## Trial status

The study was registered at https://register.clinicaltrials.gov (trial registration number: NCT04296916) on 4 March 2020. The protocol version is 2.0, dated 22/8/2020. Recruitment for the GSHM study began in April 2020, and the planned recruitment completion date is October 2021.

## Supplementary information

**Additional file 1.** SPIRIT 2013 Checklist: Recommended items to address in a clinical trial protocol and related documents.

## Data Availability

Any data required to support the protocol can be supplied on request.

## Abbreviations

BCVA: Best corrected visual acuity
BP: Blood pressure
CCT: Central corneal thickness
CI: Confidence interval
DMC: Data monitoring committee
DRQ: Data request
EDC: Electronic data collection
EMGT: Early Manifest Glaucoma Trial
EQ-5D-5L: EuroQol-5 Dimensions five-level
ETDRS: Early Treatment Diabetic Retinopathy Study
GCIPL: Ganglion cell-inner plexiform layer
GCP: Good Clinical Practices
GON: Glaucomatous optic neuropathy
GPA: Guided Progression Analysis
GSHM: Glaucoma suspects with high myopia
HM: High myopia
IOP: Intraocular pressure
IQR: Interquartile range
IRB: Institutional review board
LOCF: Last observation carried forward
LogMAR: Logarithm of the Minimum Angle of Resolution
MD: Mean deviation
NEI-VFQ-25: National Eye Institute Visual Function Questionnaire-25
OCT: Optical coherence tomography
RCT: Randomized controlled trial
RESC: Refractive Error in School Children
RNFL: Retinal nerve fiber layer
RR: Relative risk
SC: Steering committee
SD: Standard deviation
SITA: Swedish interactive threshold algorithm
UKGTS: United Kingdom Glaucoma Treatment Study
VCDR: Vertical cup-to-disc ratio
VF: Visual field
ZOC: Zhongshan Ophthalmic Center
